# Be aware, innate immune cells remember

**DOI:** 10.18632/aging.101552

**Published:** 2018-09-12

**Authors:** Niels P. Riksen, Mihai G. Netea

**Affiliations:** 1 Department of Internal Medicine, Radboud University Medical Center, Nijmegen, the Netherlands; 2 Department for Genomics and Immunoregulation, Life and Medical Sciences Institute (LIMES), University of Bonn, Bonn, Germany

**Keywords:** atherosclerosis, innate immune memory, trained immunity, aging

Aging is one of the most powerful independent risk factors for the development of atherosclerotic cardiovascular disease (CVD), and more than half of all cardiovascular deaths occur in people aged 75 years or more [1].

Among many other explanations, this could be driven by age-related changes in the immune system. Systemic inflammation contributes to atherogenesis and an increased low-level inflammation during the aging process (‘inflammaging’) has been proposed as a culprit for many age-related diseases, including CVD. Monocyte-derived macrophages are the most abundant immune cells in atherosclerotic plaques, and are key to the formation, growth, and rupture of these lesions. Our group recently reported that circulating levels of interleukin-6, interleukin-1-receptor antagonist, and interleukin-18-binding protein increase with aging [2]. In addition, monocyte production capacity for several pro-atherogenic inflammatory cytokines was higher with increasing age.

In the past few years, three novel mechanisms have been proposed to contribute to this age-related activation of the innate immune system. First, cellular senescence, a permanent arrest of cell growth, is associated with an enhanced secretion of pro-inflammatory mediators, e.g. cytokines [3]. Secondly, due to an accumulation of acquired mutations in hematopoietic stem cells that confer a competitive advantage, >10% of subjects aged over 70 years have significant amounts of mutant clones in peripheral leukocytes, which is called clonal hematopoiesis of indeterminate potential (CHIP). CHIP is associated with an increased risk for CVD because these clonal leukocytes have an increased NLRP3 inflammasome-mediated interleukin-1β secretion [4]. Thirdly, we and others have described that innate immune cells can effectively build a non-specific immunological memory that results in an increased proinflammatory phenotype, a process which is termed *trained immunity*.

Recent studies have shown that circulating monocytes and myeloid progenitor cells in the bone marrow have the intriguing capacity to reprogram towards a long-term non-specific pro-inflammatory phenotype following initial exposure to microorganisms or microbial products [5]. Although beneficial in the context of resistance against reinfections, this mechanism might be detrimental in non-infectious chronic inflammatory conditions in which myeloid cells contribute to disease progression, such as atherosclerosis. We have recently proposed this mechanism to contribute to the well-known association between acute and chronic infections and atherosclerotic CVD [6]. Interestingly, trained immunity is not only induced by microbial products, but also by endogenous sterile atherogenic stimuli such as oxidized low-density lipoprotein (oxLDL) or lipoprotein (a) [7].

It is now key to elucidate the mechanisms that drive trained immunity, since this might reveal novel targets for preventive drug treatment. We have previously reported that reprogramming of intracellular metabolic pathways and epigenetic reprogramming are two fundamental processes in trained immunity, that are closely intertwined [7]. Briefly, trained monocytes and macrophages have avid glycolytic capacity and an increased glutaminolysis that feeds into the Krebs cycle. Intermediate metabolites from these pathways co-regulate the activity of epigenetic enzymes, which culminates in long-term activation of pro-inflammatory gene transcription by enrichment of activating histone marks and increase chromatin accessibility. Importantly, these adaptations not only occur in circulating mature monocytes, but also in hematopoietic stem and progenitor cells in the bone marrow niche, which explains the observation that trained monocytes remain present in the circulation after initial stimulation for more than three months [8].

In conclusion, in the past five years, immunological research has flourished in identifying novel mechanisms that modulate immune cell function. Because these mechanisms involve gradual accumulation of somatic mutations (CHIP) or the long-term adaptation to environmental cues (trained immunity), they are of particular interest for age-related disease such as CVD. Further elucidation of these pathophysiological processes, in combination with innovative drug development and drug-delivery systems that enable to target specific cells types, might provide completely novel classes of drugs to prevent atherosclerotic cardiovascular disease.
